# Supplementary material for: Qualitative insights into reasons for missed opportunities for vaccination in Kenyan health facilities
Source: PLoS One. 2020 Mar 30;15(3):e0230783. doi: 10.1371/journal.pone.0230783 (PMC7105087; doi:10.1371/journal.pone.0230783)
Supplement: S1 File — (PDF) [file pone.0230783.s001.pdf]

MINISTRY OF HEALTH

Telegrams: "FAMHEALTH', Nairobi
Telephone:0202721057
Email: head.epi@gmail.com
All correspondence should be addressed to the Head
When replying please quote

DIVISION OF FAMILY HEALTH
MBAGATHI ROAD (OLD)
P.O BOX 43319 – 00100
NAIROBI

MOH/NVIP/04/9/VOL.1

14th January, 2020

The Managing Editor,
PLOS One Journal

Re: PONE-D-19-31527 (Assessment of missed opportunities for vaccination in Kenyan health facilities, 2016)- Clarification of ethical review for standard program reviews in Kenya

Greetings!

The Missed Opportunities for Vaccination assessments in Kenya were considered and conducted as program reviews, similar to EPI reviews and vaccine post-introduction evaluations. As such, the Ministry of Health Kenya does not subject these protocols to ethical board review. As the Manager of the National Vaccines and Immunization Program of the Ministry of Health Kenya and a co-author on this manuscript, I handled the internal informational process. It was internally confirmed at the Ministry of Health Kenya as a program assessment, that no formal review was needed and it was exempt from Institutional Review Board.

The study team also included a verbal consent procedure before administering surveys to ensure participants had the opportunity to understand the assessment procedure and to decline participation. They were informed that participation was voluntary, they could leave the assessment at any time, no personally identifiable information would be collected, and could choose to not answer questions without repercussion. All data collectors obtained oral consent from all participants. We approved the verbal consent procedure at the Ministry of Health Kenya.

Thank you.

Dr. Collins W. Tabu
HEAD, DIVISION OF NATIONAL VACCINES & IMMUNIZATION PROGRAM,KENYA

SGS UKAS
ISO 9001:2015
Certified
