# Supplementary material for: Qualitative insights into reasons for missed opportunities for vaccination in Kenyan health facilities
Source: PLoS One. 2020 Mar 30;15(3):e0230783. doi: 10.1371/journal.pone.0230783 (PMC7105087; doi:10.1371/journal.pone.0230783)
Supplement: S3 File — (PDF) [file pone.0230783.s003.pdf]

FGD Guide for Mothers/Caregivers

Opening Questions

1. Let's start with some introductions. Can we go around the circle and everyone say their name and the ages of their children? You can feel free to introduce yourself with any name you choose.

2. What are some health problems that affect children in this community?

3. How do you prevent your children from being affected by these health problems?

    a. Probe: If vaccination is not mentioned, *what about vaccination*?

Key Questions

General Vaccination

1. How does the community feel about childhood vaccination?

2. What can you tell us about childhood vaccination services in this community?

    a. Probe: Where do you get vaccination services for children in this community?

    b. Probe for their levels of satisfaction with the vaccination services they receive from the private and/or public clinics/hospitals (e.g. How do you feel about the vaccination services you receive from these locations?)

    c. Probe for reasons for their satisfaction or dissatisfaction (e.g. Why?)

3. How can these services be improved?

Vaccine Compliance

4. In Kenya, as you may be familiar with, the national programme sets a vaccination schedule. Do children often receive their vaccines following this schedule?

5. Many children do NOT receive all their vaccines on time, as scheduled in the national programme. What are some of the reasons children DO NOT receive all their recommended vaccines on time?

    a. Probe: What are some of the reasons children DO receive all their recommended vaccines on time?

6. What will be your suggestions for helping children to receive all their recommended vaccines according to the schedule?

Missed Opportunities

7. In some clinics, the health workers do NOT always give children all the vaccines they need. What are reasons **some health workers** may not be willing or able to give children all their recommended vaccines on time, when they visit the clinic/hospital?

8. Some children receive some, but not all the vaccines they need. What are some reasons **mothers/caregivers** may not be willing or able to ensure that their children receive all their recommended vaccines on time when they visit the clinic/hospital?

9. In some cases, children who visit health facilities, for different reasons, still do not get all the needed vaccines. What are some **recommendations** for ensuring that children receive all their recommended vaccines, whenever they have the opportunity of visiting a clinic/hospital for any reason? (They may be visiting for immunization, nutrition, treatment of other ailments, or accompanying an adult to the clinic/hospital)?

Closing questions

1. Are there additional recommendations that you would like to share at this time?

2. Does anyone else have anything they would like to add?
