# Supplementary material for: Qualitative insights into reasons for missed opportunities for vaccination in Kenyan health facilities
Source: PLoS One. 2020 Mar 30;15(3):e0230783. doi: 10.1371/journal.pone.0230783 (PMC7105087; doi:10.1371/journal.pone.0230783)
Supplement: S4 File — (PDF) [file pone.0230783.s004.pdf]

KII Guide

Opening Questions

1. Let's start with some introductions. Can you tell me a little bit about yourself and work here at [health facility]?

2. What are some health problems that affect children you see at [health facility]?

3. How do you help prevent children from being affected by these health problems?
   a. *Probe:* If vaccination is not mentioned, *what about vaccination?*

Key Questions

Vaccination Services

1. What can you tell us about vaccination services at this [health facility]?

2. What are some challenges to delivering vaccination services at [health facility]?

   a. Probe for their levels of satisfaction among caregivers with the vaccination services children receive.

3. How can vaccination services at [health facility] be improved?

Vaccine Compliance

4. In Kenya, as you are familiar with, the national programme sets a vaccination schedule. Do children often receive their vaccines following this schedule?

5. Many children DO NOT receive all their vaccines on time, as listed on the national schedule. What are some of the reasons children DO NOT receive all their recommended vaccines on time when they visit the clinic/hospital?

   a. Probe: What are some of the reasons children DO receive all their recommended vaccines on time?

6. What will be your suggestions for helping children to catch up with their vaccinations, if needed?

KII Guide

Missed Opportunities

7. In some clinics, the health workers DO NOT always give children all the vaccines they need. What strategies, if any, can the ministry employ to improve on the number of children that receive their recommended vaccinations on time?

    a. Probe: Strategies other critical entities can employ?

8. Some children receive some, but not all the vaccines they need. How can we ensure that any child who comes to the clinic/hospital is up-to-date with their vaccination?

9. In some cases, children who visit health facilities, for different reasons, still do not get all the needed vaccines. (They may be visiting for immunization, nutrition, treatment of other ailments, or accompanying an adult to the clinic/hospital). What are the possible barriers to implementing any of these strategies to reduce missed opportunities?

    a. Probe: Possible solutions to any barriers that have been mentioned

Closing Questions

1. Are there additional recommendations that you would like to share at this time?

2. Does anyone else have anything they would like to add?
