# Supplementary material for: Qualitative insights into reasons for missed opportunities for vaccination in Kenyan health facilities
Source: PLoS One. 2020 Mar 30;15(3):e0230783. doi: 10.1371/journal.pone.0230783 (PMC7105087; doi:10.1371/journal.pone.0230783)
Supplement: Attachment — Submitted filename: Reviewer Comments_2.6.20v2.docx [file pone.0230783.s005.docx]

| **Qualitative Insights into Reasons for Missed Opportunities for Vaccination in Kenyan Health Facilities** | | |
| --- | --- | --- |
| **#** | **Reviewer Comments** | **Author Response** |
| **Editor Comments** | | |
| 1 | Please ensure that your manuscript meets PLOS ONE's style requirements, including those for file naming. | We have updated necessary sections and files to ensure that our manuscript meets PLOS ONE’s style and file naming requirements. |
| 2 | Please specify in your ethics statement:  a) whether the ethics committee approved the verbal/oral consent procedure,  b) why written consent could not be obtained, and  c) how verbal/oral consent was recorded. | We have included this information in the ethics statement.   a) Yes, the verbal consent procedure was approved by the Ministry of Health Kenya b) Written consent was not required by the Ministry of Health Kenya, as this was assessed as a programme review. Please find attached as S1 File an official letter from the ministry affirming this fact. c) Consent was recorded by the facilitator or interviewer on the FGD or in-depth interview guide |
| 3 | Please include in your Methods section the date ranges over which you recruited participants to this study. | We have added the dates of field work to the Methods section under “Data collection.” |
| 4 | Please include additional information regarding the survey or questionnaire used in the study and ensure that you have provided sufficient details that others could replicate the analyses. If you developed and/or translated a questionnaire as part of this study and it is not under a copyright more restrictive than CC-BY, please include a copy, in both the original language and English, as Supporting Information. | We have included more information regarding the focus group discussion and in-depth interview guides.  The generic guides can be found on the WHO MOV website (included in the references). We have also included a copy of the guides used in Kenya as S2-S4 Files. |
| 5 | We note that one or more of the authors are employed by a commercial company: John Snow, Inc. Country Office, Nairobi, Kenya.  a.     Please provide an amended Funding Statement declaring this commercial affiliation, as well as a statement regarding the Role of Funders in your study. If the funding organization did not play a role in the study design, data collection and analysis, decision to publish, or preparation of the manuscript and only provided financial support in the form of authors' salaries and/or research materials, please review your statements relating to the author contributions, and ensure you have specifically and accurately indicated the role(s) that these authors had in your study. You can update author roles in the Author Contributions section of the online submission form. We note that one or more of the authors are employed by a commercial company: John Snow, Inc. Country Office, Nairobi, Kenya.  b. Please also provide an updated Competing Interests Statement declaring this commercial affiliation along with any other relevant declarations relating to employment, consultancy, patents, products in development, or marketed products, etc. | JSI has two arms – a commercial and a non-commercial entity. The co-author of this paper was affiliated with the non-commercial entity (JSI Research and Training Institute) and was funded by a USAID grant, under the Maternal and Child Survival Program. We have updated his affiliation to reflect this. |
| 6 | We note that Figure #1 in your submission contain [map/satellite] images which may be copyrighted. All PLOS content is published under the Creative Commons Attribution License (CC BY 4.0), which means that the manuscript, images, and Supporting Information files will be freely available online, and any third party is permitted to access, download, copy, distribute, and use these materials in any way, even commercially, with proper attribution. For these reasons, we cannot publish previously copyrighted maps or satellite images created using proprietary data, such as Google software (Google Maps, Street View, and Earth). | We have removed Figure #1 and added text to the methods describing the counties selected. |
| **Reviewer #1** | | |
| 7 | Background:  Line 45: Include more information about immunization coverage in Kenya and the factors that have been identified to predict low immunization coverage in the country. | We have added additional information to the background focusing on past work conducted around missed opportunities for vaccination (MOV) in Kenya to provide country context and set the scene for how this MOV assessments fills a gap. |
| 8 | Background:  Line 47 – 50: To improve flow, you may consider moving this part to the last paragraph of the background section. | We have moved this to the last paragraph of the background section. |
| 9 | Methodology:  The term “cross-sectional” is commonly associated with quantitative studies. Given that the philosophical assumptions of this class of studies is markedly different from qualitative, I’d suggest that it should be avoided here. | We have removed “cross-sectional.” |
| 10 | Methodology:  The rationale for using grounded theory in this manuscript is debatable. MOV is already a pre-existing concept. For this reason, the goal of the study was not to develop new theories (and a theoretical framework) about MOV. In my opinion, this study leans more towards a critical realism because of the causal-explanatory approach that was adopted to describe factors that are responsible for MOV. Moreover, the study builds on already existing knowledge by exploring Kenya’s context and identified factors that are relatively similar to those identified in existing literature. Overall, there is a need to extensively and convincingly justify the methodological paradigm that is being adopted in the study design sub-section. | On further review, we agree that our study does not fully align with standard grounded theory, as we did not aim to develop new theories. In fact, the qualitative study we have reported is part of a larger MOV assessment and the conclusions/interventions are not drawn from only qualitative data, but the triangulation of all the data sources. While critical realism might be a better alternative, we have opted to edit the study design sub-section to de-emphasize alignment with any established paradigms. |
| 11 | Methodology:  Line 80: Include a sub-section on reflexivity | We have included this under “Field team recruitment and training.” |
| 12 | Methodology:  Line 100: Please remove “sample size” | Removed. |
| 13 | Methodology:  Line 101: What were the criteria for this purposive selection? How do the counties differ from each other? Why 10? | We have outlined the criteria for purposive county selection including representing various geographic regions across the country and vaccination performance levels. We have included that logistical considerations also played a role and 10 counties were selected as that was the maximum feasible spread and number thought to provide a snapshot of the situation of MOV in Kenya. In addition, the 10 study counties represent more than a fifth of the counties in Kenya. |
| 14 | Methodology:  What were the criteria used to stop data collection? | Because all 10 unique field teams were simultaneously collecting data from the field, it was impossible to review the data in real time to identify saturation. Under Methodology in section “Participant selection,” we discuss that we set a goal for field teams (1 FGD with caregivers, 1 FGD with health workers, and 4 IDIs with key informants). Qualitative data collection was complete when the field team had reached this goal. |
| **Reviewer #2** | | |
| 15 | This paper presents a qualitative study which examined the reasons for missed opportunities for vaccinations in Kenyan Health facilities. I do believe this paper would be of interest to readers of PLOS ONE however, the following issues outlined below need to be addressed. | Thank you for your review and for acknowledging the potential contribution of our paper. We have also addressed the concerns you raised. |
| 16 | Background:  Lines 47-50: can this be moved to the final paragraph of the introduction? | We have moved this to the last paragraph of the background section. |
| 17 | I think more references to other studies conducted in Kenya on vaccine uptake needs to be added in the introduction. That way it paints a clearer picture of the research gap as well as how this study fits into the literature. I don't quite follow at the moment and it is not quite clear. | We have added additional information to the background focusing on past work conducted around MOV in Kenya to provide country context and set the scene for how this MOV assessments fills a gap. |
| 18 | Methods:  Can you clarify what type of thematic analysis was used? It seems like an inductive approach was used but this needs to be mentioned in the methods so that it is clear to the reader. | We have clarified that we conducted inductive thematic analysis. |
| 19 | Discussion:  This is currently very long and needs to be streamlined. A lot of time is spent regurgitating the results and little time is spent engaging with the literature.  Did you look for any differences and similarities within your sample? Perhaps also look at other studies that have examined vaccine uptake in Kenyan populations globally? And see if some of the issues identified are the same or different? Obviously there would be a huge distinction between those in low income vs. high income countries but this might add some interesting nuances. | We have streamlined the discussion.  Although we included counties of varying demographics and immunization coverage performance, we did not look for differences and similarities within our sample as this assessment is meant to be a low-resource approach to identify easily-implementable but impactful activities across Kenya as a whole. We did identify areas that were more specific to the particular groups of study populations (health workers, caregivers, key informants) and have identified those differences throughout the manuscript. Additionally, we have highlighted other studies in the discussion that focused more on specific gaps we found (e.g. increased education and positive impact on vaccine uptake, effect of rumors and vaccine hesitancy, multi-dose vial practices, etc.). |
